# Tailoring Mn_3_O_4_ nanoparticle morphology *via* polyethylene glycol mediation for aqueous rechargeable zinc-ion batteries

**DOI:** 10.1039/d6ra01648k

**Published:** 2026-05-28

**Authors:** Habiba Jahan, Mohammad Abu Yousuf, Nusrat Tazeen Tonu, Parbhej Ahamed, Ahammad Musa, Md. Sadiqul Islam Sheikh

**Affiliations:** a Department of Chemistry, Khulna University of Engineering & Technology Khulna-9203 Bangladesh yousuf@chem.kuet.ac.bd; b Chemistry Discipline, Khulna University Khulna-9208 Bangladesh

## Abstract

Aqueous rechargeable zinc-ion batteries (ARZIBs) are highly promising next-generation energy storage systems, yet their deployment is often hindered by sluggish cathode diffusion kinetics. Herein, morphology-tuned Mn_3_O_4_ nanoparticles were successfully synthesized *via* a facile, eco-friendly route employing polyethylene glycol (PEG 3350) as a structure-directing soft template. FESEM and TEM confirmed the formation of uniform spherical morphology. The engineered nanostructure exhibited an optical band gap of 3.87 eV and enhanced surface hydrophilicity, which significantly optimized electrolyte–electrode interfacial contact. When evaluated as an ARZIBs cathode, the cell demonstrated highly reversible Zn^2+^/H^+^ co-intercalation chemistry with a dominant diffusion-controlled pseudo-capacitive charge storage mechanism. Driven by initial phase transformation and electrochemical self-activation, the cathode delivered a remarkable peak specific discharge capacity of 274.82 mAh g^−1^ at a current density of 0.1 A g^−1^ and maintained a capacity retention of 76.12% after 500 cycles. EIS confirmed low initial charge-transfer resistance, validating the superior kinetics of the morphology-tailored cathode. This study highlights green morphology engineering as an effective paradigm to construct high-performance manganese-based cathodes for advanced ARZIBs applications.

## Abstract 2

PEG-assisted synthesis of Mn_3_O_4_ nanoparticle as high-performance cathode for aqueous rechargeable Zn-ion battery.

## Graphical abstract

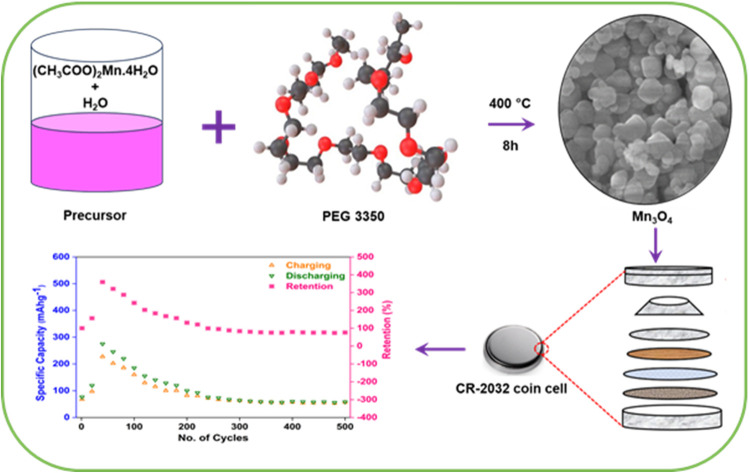

## Introduction

With the escalating global demand for sustainable and clean energy, the search for efficient, low-cost, and eco-friendly energy storage devices and the rapid adoption of renewable energy sources and the electrification of transportation have become paramount.^1^ Lithium-ion batteries (LIBs) have long been the cornerstone of energy storage due to their high energy density (170–250 Wh kg^−1^), high specific power (200–1000 W kg^−1^), and a long cycle life (up to 3000 cycles).^2,3^ However, several drawbacks such as high cost, resource scarcity, and safety concerns related to flammable organic electrolytes, limiting their application in large-scale storage.^4^ Lithium-sulfur (Li–S) batteries are considered highly promising next-generation energy storage systems because of their remarkable theoretical specific capacity (1675 mAh g^−1^) and high energy density (2600 Wh kg^−1^), which are much greater than those of conventional lithium-ion batteries. Nevertheless, their practical application remains limited due to critical issues associated with both the sulfur cathode and lithium metal anode.^5^ Solid-state lithium batteries are viewed as a viable pathway to address the safety and performance constraints of conventional lithium batteries, due to their non-flammable solid electrolytes, expanded electrochemical stability window, and capability for higher energy density.^6^ At present, the energy density of lithium-ion batteries (LIBs) is largely restricted by the cathode materials, prompting the search for alternative energy storage technologies.^7^ Similarly, lead-acid batteries pose environmental hazards due to toxic components like lead and acid waste.^8^ Therefore, it is essential to develop alternative secondary batteries capable of meeting growing energy demands. Among the available options, potassium-ion batteries have emerged as encouraging candidates for energy storage due to their abundant resources, cost-effectiveness, and low standard potential (−2.93 V).^9^ Complementary energy storage technologies, such as supercapacitors utilizing conjugated polymers, also contribute to sustainable energy solutions by providing high power density and rapid charge–discharge capabilities.^10^ In contrast, aqueous rechargeable zinc-ion batteries (ARZIBs) have emerged as a promising alternative, offering advantages like high theoretical capacity (820 mAh g^−1^), high volumetric capacity (5855 mAh cm^−3^), cost-effectiveness (≈1.35 USD$/lb) of Zn foil anode, intrinsic safety and ionic conductivity (1 S cm^−1^) of the aqueous electrolytes, and environmental compatibility.^11,12^ However, the performance of ARZIBs is critically dependent on the cathode material, which often suffers from low capacity, poor cycling stability, and sluggish kinetics.^13,14^ Various cathode materials, including Prussian blue analogues, manganese-based compounds, vanadium-based materials, and organic species, have been extensively investigated for aqueous ARZIBs. Metal–organic framework (MOF)-derived materials have recently attracted significant attention in energy storage applications owing to their high specific surface area, efficient mass transport pathways, and abundance of accessible electroactive metal sites.^15^ Among the various cathode candidates, manganese oxides (Mn_*x*_O_*y*_) are particularly attractive due to their multivalent nature, which enables high theoretical capacities, as well as their low cost and environmental benignity.^16,17^ Mn predominantly exists as Mn^3+^ and Mn^4+^. Mn^3+^, with a high-spin electronic configuration and a single electron in the eg* orbital, induces Jahn–Teller distortion of the octahedral structure.^18^ This local distortion leads to irreversible structural changes and rapid capacity fading. Considerable efforts have been made to enhance structural stability and prevent metal dissolution through strategies such as surface modification.^19^ In this context, the phase field method (PFM) has emerged as a powerful computational approach for predicting nano- and mesoscale microstructural evolution during material processes, providing deeper insight into structural instability and degradation mechanisms.^20^

Mn_3_O_4_ known as hausmannite, is a mixed-valence manganese oxide (Mn^2+^Mn^3+^_2_O_4_) with a spinel structure.^21^ However, a major challenge lies in its poor intrinsic electronic conductivity and the significant volume expansion it undergoes during the insertion and extraction of large hydrated zinc ions. These factors lead to sluggish reaction kinetics and rapid capacity fade, limiting its long-term cycling stability.^22^ Tailoring the morphology as well as nano-structuring has been identified as a viable strategy to mitigate these issues.^23^ The possible limitations of Mn_3_O_4_ cathodes for long-term applications, including Mn dissolution, low intrinsic conductivity, structural instability, and sluggish ion diffusion, can be effectively addressed through several advanced strategies reported in recent literature. Surface coating or protective interphase engineering using TiO_2_, carbon, or conductive polymers can suppress Mn dissolution and stabilize the electrode/electrolyte interface; for example, Yang *et al.* (2025) reported that a TiO_2_ interphase on Mn-based cathodes significantly enhanced interfacial stability and delivered 87.6% capacity retention after 1000 cycles at 10 A g^−1^. Cation doping with Ce^3+^, Co^2+^, Fe^3+^, or Zn^2+^ can mitigate Jahn–Teller distortion, generate oxygen vacancies, and improve conductivity, thereby enhancing structural durability and cycling reversibility.^24^ Electrolyte optimization, particularly by introducing Mn^2+^ additives (*e.g.*, MnSO_4_) into ZnSO_4_ electrolytes, can reduce irreversible Mn loss by shifting the dissolution/deposition equilibrium and suppressing byproduct formation. In addition, morphology and defect engineering, such as constructing hierarchical or hollow nanostructures with oxygen vacancies or pre-inserted ions, can buffer volume variation and shorten Zn^2+^ diffusion pathways.^25^ Furthermore, forming composites with highly conductive matrices such as graphene, carbon nanotubes (CNTs), MXene, or carbon nanofibers can greatly improve charge transport and mechanical integrity, resulting in superior long-term stability. A simple and effective method for achieving this morphological control may be developed through the use of polyethylene glycol (PEG). PEG is a non-toxic and versatile polymer that acts as a mediating agent during the nanoparticle synthesis.^26^ It functions as a soft template, with its polymer chains adsorbing onto specific crystal facets of the growing nanoparticles. The presence of PEG facilitates the formation of porous and high-surface-area architectures, influences the anisotropic growth which improve the Zn^2+^ ion diffusion and show high electrochemical performance.^27–29^ It not only regulates particle size and prevents agglomeration but also contributes to surface functionalization, potentially improving wettability and ion transport kinetics.^14^ Furthermore, the decomposition of PEG during calcination can create mesoporous frameworks, which provide multiple channels for Zn^2+^ ion diffusion and increase the number of active redox sites.^29,30^ Compared to gelatin 31, cellulose,^1^ and rice powder,^32^ PEG offers better solubility control, tunable molecular weight, and thermal stability, making it an ideal candidate for scalable Mn_3_O_4_ synthesis. The application of green templating strategies for Mn_3_O_4_ synthesis is not new. Tonu *et al.* demonstrated the use of bloom gelatin as a biopolymeric template for synthesizing Mn_3_O_4_, resulting in coin cells with a specific discharge capacity of 219.33 mAh g^−1^ and high coulombic efficiency. Their study confirmed that soft templates significantly influence particle dispersion, crystallinity, and electrochemical behavior.^31^ In another work, cellulose-supported CuO cathodes fabricated by Hassan *et al.* (2025) exhibited high performance and excellent cycling stability, emphasizing the importance of eco-friendly templating methods in battery research. The resulting coin cells exhibited a specific discharge capacity of 253.03 mAh g^−1^ at a current density of 100 mA g^−1^ and a coulombic efficiency of 98.28% after 800 cycles.^1^ Similarly, Tonu *et al.* synthesized bixbyite Mn_2_O_3_ microdice *via* an ultrasonic-assisted reverse micelle method for ARZIB cathodes. The material exhibited a 293.59 mAh g^−1^ discharge capacity at 0.1 A g^−1^, 90.35% capacity retention, and 98.44% coulombic efficiency after 1000 cycles, attributed to its porous structure and high crystallinity.^33^ Li *et al.* employed a manganese glycerate template to synthesize MnO_2_ microspheres for ZIBs. The material showed a specific capacity of 259 mAh g^−1^ at 0.5 A g^−1^, with enhanced ion transport, demonstrating the efficacy of templates in battery applications.^34^

In this work, PEG 3350 was strategically employed as a structure directing template to finely tune the morphology of Mn_3_O_4_, successfully suppressing random crystal growth to yield structured spherical type nanoparticles with optimized electrochemical kinetics. When evaluated as a cathode material in CR-2032 coin-type ARZIBs, the engineered nanostructure exhibited significantly enhanced electrochemical performance, including a superior specific discharge capacity, outstanding rate capability, and long-term cycling durability.

## Materials and methods

### Chemicals and instruments

Manganese(ii) acetate tetrahydrate, (CH_3_COO)_2_Mn·4H_2_O CAS No. 6156-78-1, was purchased from Sigma-Aldrich (USA, purity 99%). Extra pure polyethylene glycol 3350 (PEG) CAS No. 25322-68-3 was collected from Incepta Pharmaceuticals Limited (Bangladesh). Stainless steel (SS) substrates (304 grades) of thickness *t*-0.01× w-100 mm, zinc foil, polyvinylidene fluoride (PVDF), *N*-Methyl-2-pyrrolidone (NMP) and carbon black (C-black) were used for the preparation of CR-2032 coin cell battery. The whole experiment was carried out using deionized (DI) water; prepared by deionized water system (Model: WDI-15, Human Science, South Korea) available in the laboratory. The structure and morphology of prepared samples were investigated using FESEM machine (JSM-7610F, Jeol Japan). X-ray diffraction analysis was done using X-ray diffractometer (XRD: Bruker, D2 PHASER, Germany) with Cu Kα radiation at 2*θ* from 10 to 80°. FTIR was done using FTIR spectrometer (IR Tracer-100, Shimadzu Corporation, Japan). Solid state UV visible spectroscopy was applied to find out the optical band gap and recorded within the range of 200–1000 nm using a UV spectrophotometer (UV-1800, Shimadzu Corporation, Japan). Raman spectroscopy was conducted using a DXR Smart Raman spectrometer from Thermo Fisher Scientific (USA), equipped with a 780 nm excitation laser and a full-range grating. The elemental constituents and manganese valency were characterized through X-ray photoelectron spectroscopy (XPS; L-Alpha, Thermo Scientific, UK). The prepared materials shape and crystal planes were evaluated using transmission electron microscopy (TEM) (JEM 2100 Plus Electron Microscope), JEOL, Japan). The wettability of the material was evaluated by measuring the contact angle using an Ossila contact angle goniometer (L2004A1). CV, EIS, and BCD were performed *via* Biologic (SP 300) potentiostat.

### Preparation of Mn_3_O_4_ using polyethylene glycol (PEG) as template

**Fig. 1:**
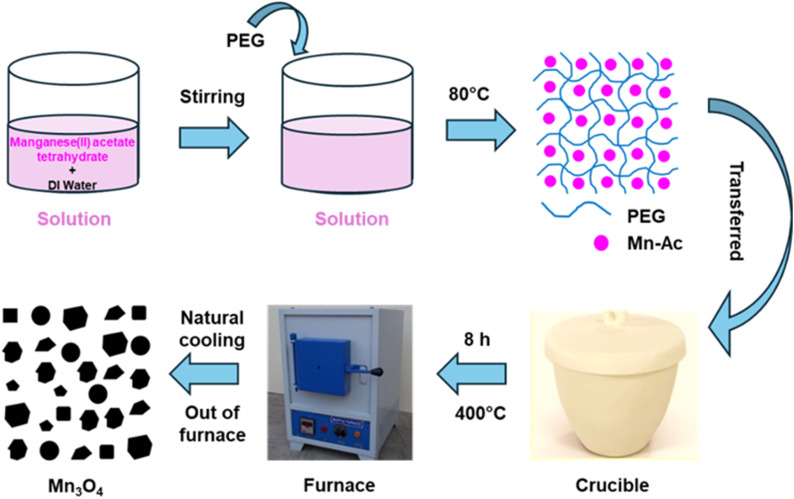
Preparation of Mn_3_O_4_ (schematic diagram).

5 mL of deionized water and 1.0 g of manganese(ii) acetate tetrahydrate were added to a crucible. The mixture was stirred until complete dissolution was achieved. A weight ratio of 1 : 0.5 between manganese(ii) acetate tetrahydrate and PEG was then maintained by adding 0.5 g of PEG to the mixture. Stirring continued until a macroscopic homogeneous mixture was formed. The precursor mixture (manganese(ii) acetate tetrahydrate + PEG 3350) was dried at 80 °C to form a homogeneous melt, then transferred to a muffle furnace and calcined at 400 °C for 8 h in air. The heating ramp rate was 5 °C min^−1^, and the sample was allowed to cool naturally inside the furnace to room temperature after the dwell time. Finally, the black color powder was formed as the desired product and labelled as P1M1. The same procedure was repeated for weight ratios of 1 : 1 and 1 : 2 (manganese(ii) acetate tetrahydrate to PEG), producing samples labelled as P2M1 and P3M1, respectively. These conditions were kept identical for all samples (M1, P1M1, P2M1, and P3M1). A manual mortar and pestle were used to grind the resulting powders before they were used in additional experiments. Fig. 1 Showed a schematic illustration of the product synthesis process. Another experiment was conducted without the addition of PEG, with simply deionized water and manganese(ii) acetate tetrahydrate. The synthesized product was labelled as M1.

One possible explanation for the creation of Mn_3_O_4_ particles is as follows.^35^
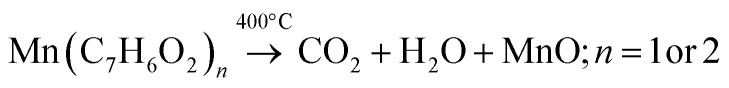

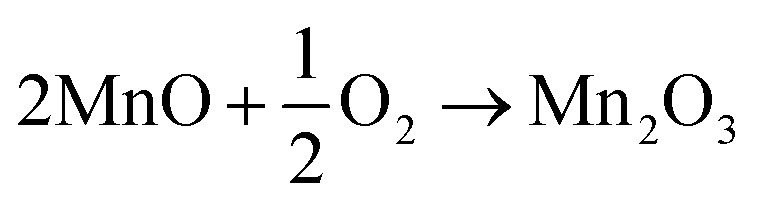
Mn_2_O_3_ + MnO → Mn_3_O_4_

### Coin cell fabrication

The synthesized P3M1 (as it contains smallest particle sizes and uniformity) was blended with conductive C-black and PVDF in a weight ratio of 8 : 1 : 1, using NMP as solvent. The resulting slurry was prepared using a mortar and pestle, then coated onto a stainless steel (SS) foil uniformly to form a thin cathode layer. The coated foil was dried at 70 °C to ensure full solidification before being processed by a battery dice cutting machine. It was then punched into 1.44 cm^2^ squares to form the cathode for a CR-2032 coin cell. The average mass loading of active material (Mn_3_O_4_) was 8 mg cm^−2^. CR-2032 coin cells were assembled in ambient laboratory air. The cathode was the prepared Mn_3_O_4_ electrode, the anode was zinc foil (thickness ∼0.5 mm, length 1.3 cm), and the separator was a commercial Whatman filter paper. The electrolyte was a 2 M ZnSO_4_ aqueous solution. Approximately 200 µL of ZnSO_4_ was added per cell before crimping and no Mn^2+^ additive was used. To assemble the coin cell, the components including the metal casing, prepared cathode, separator, anode, spacer, and spring were carefully layered in order and sealed using a crimping machine.

### Physical characterization

#### FTIR analysis

**Fig. 2:**
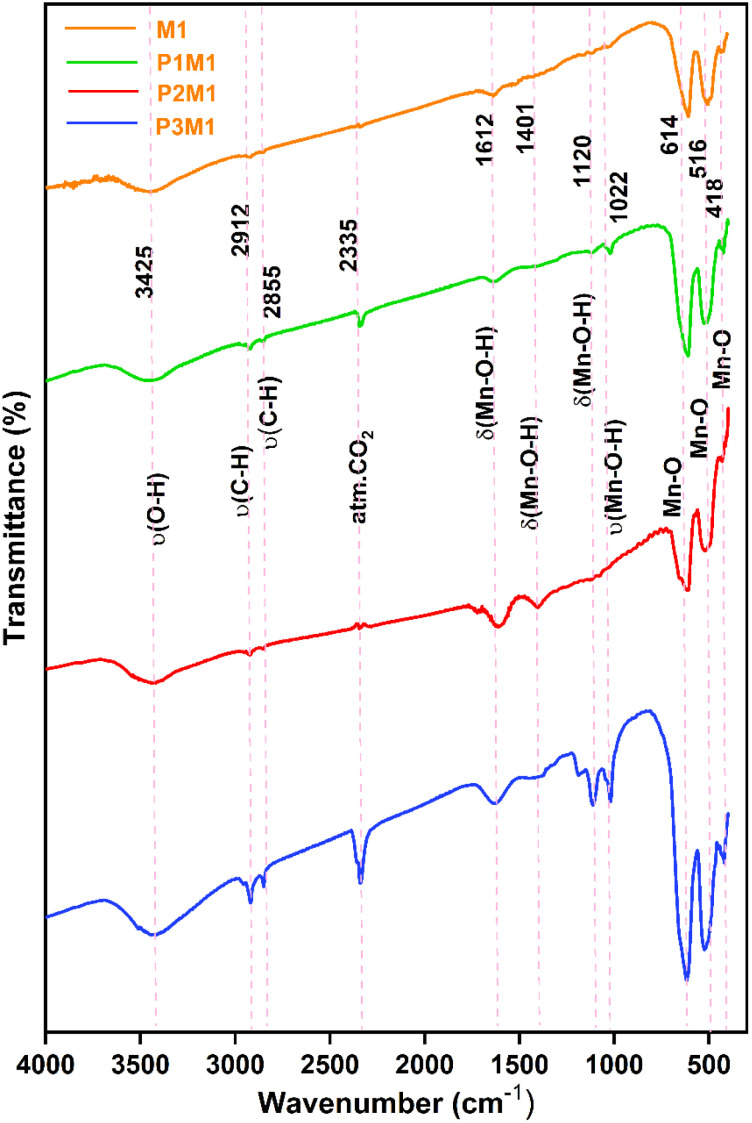
FTIR spectra of prepared samples.

To determine the chemical bonding characteristics of the synthesized samples, FTIR analysis was carried out over the spectral range of 300–4000 cm^−1^. The FTIR spectra, as shown in Fig. 2, identified three prominent peaks within the 450–650 cm^−1^ region. The band at 614 cm^−1^ was associated with Mn–O stretching vibrations in tetrahedral sites, while the peak at 516 cm^−1^ corresponded to distortion vibrations of Mn–O in an octahedral configuration. The third peak, appearing at 418 cm^−1^, was attributed to vibrations of Mn^3+^ species located in octahedral position of Mn_3_O_4_.^36^ A weak absorption at 1022 cm^−1^ was assigned to Mn–O–H stretching vibrations. In FTIR spectra of Mn_3_O_4_ materials, O–H bending vibrations often overlapped with other vibrational modes involving Mn atoms. Hence it was conferred that peaks at 1120, 1401, and 1612 cm^−1^ were attributed to O–H bending vibrations combined with Mn atoms.^37^ The band at 2335 cm^−1^ was ascribed to adsorbed atmospheric CO_2_, whereas absorptions at 2855 and 2912 cm^−1^ were related to C–H stretching of –CH_2_– groups, likely due to residual species.^1^ The broad absorption bands around 3425 cm^−1^ appeared due to O–H stretching in absorbed water molecules. These FTIR results confirmed the formation of Mn_3_O_4_. Furthermore, with the increasing amount of PEG, the line intensity of Mn_3_O_4_ decreased as the wavenumber increased, which implied its enhanced crystalline and semiconducting behavior.

#### FESEM analysis

**Fig. 3:**
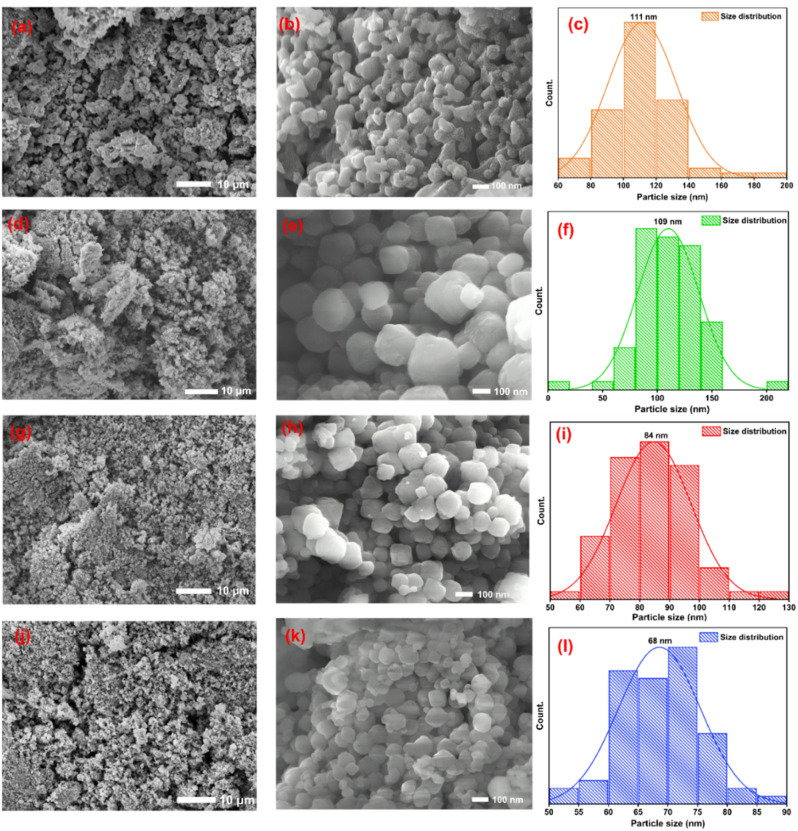
FESEM image of M1(without PEG template) at a scale bar of (a) 10 µm, (b) 100 nm & (c) histogram of particle size distribution. FESEM image of P1M1(with PEG template) at a scale bar of (d) 10 µm, (e) 100 nm & (f) histogram of particle size distribution. FESEM image of P2M1 at a scale bar of (g) 10 µm, (h) 100 nm & (i) histogram of particle size distribution. FESEM image of P3M1 at a scale bar of (j) 10 µm, (k) 100 nm & (l) histogram of particle size distribution.

**Fig. 4:**
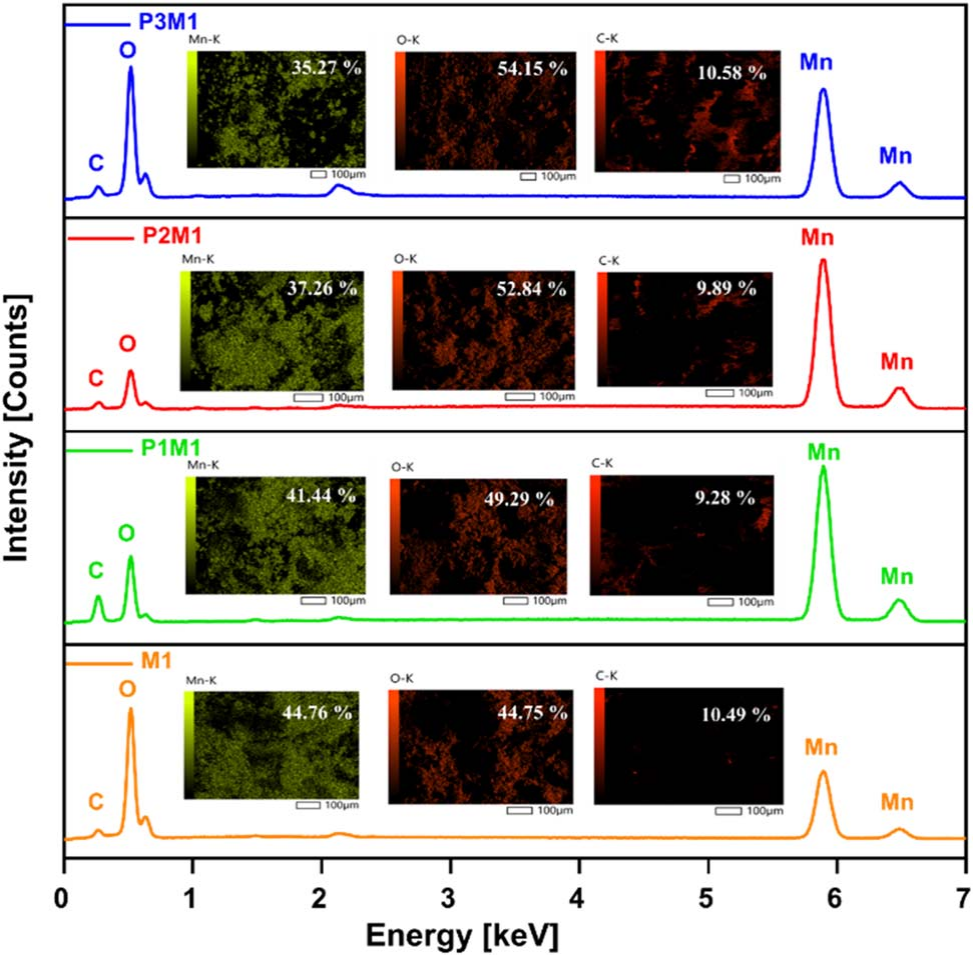
EDX spectra and elemental mapping of Mn, O, and C in M1, P1M1, P2M1, and P3M1 samples (inside showing atom % of Mn, O, and C).

The surface morphology and particle size distribution of the synthesized samples were investigated using FESEM micrographs at low magnification (10 µm scale) and high magnification (100 nm scale), as presented in [Fig. 3(a–l)]. The low-magnification FESEM images [Fig. 3(a), (d), (g) and (j)] revealed the overall surface texture and agglomeration behavior, whereas the high-magnification images [Fig. 3(b), (e), (h) and (k)] provided detailed insight into particle shape and grain distribution. In M1 [Fig. 3(a) and (b)], the particles appeared as tightly packed clusters with irregular sizes and seemed to be in peanut shapes. The absence of a mediating agent resulted in uncontrolled growth and noticeable agglomeration. With the introduction of PEG in P1M1 [Fig. 3(d) and 4(e)], the particles became comparatively more uniform and less agglomerated, although some irregularly shaped grains were still observed. A further increase in PEG concentration in P2M1 [Fig. 3(g) and (h)] resulted in a more homogeneous surface morphology with comparatively well-defined particles. Among all samples, P3M1 [Fig. 3(j) and 4(k)] exhibited the finest microstructure with more regular and uniform spherical particles, indicating improved growth control in the presence of the highest PEG concentration. Particle size histograms derived from ImageJ analysis were shown in Fig. 3(c), (f), (i) and (l). For statistical reliability, approximately 70 particles were measured for each sample. The average particle sizes were 111 ± 28 nm, 109 ± 21 nm, 84 ± 12 nm and 68 ± 7 nm for M1, P1M1, P2M1 and P3M1, respectively.^38^ Overall, increasing PEG concentration resulted in a progressive decrease in average particle size from 111 nm to 68 nm, demonstrating more controlled nucleation and growth.

#### EDX analysis

EDX was utilized to investigate the elemental composition and stoichiometry of the synthesized specimens. The EDX spectra, as shown in Fig. 4, displayed prominent peaks that corresponded to manganese (Mn) and oxygen (O), along with a minor peak that indicated the presence of carbon (C). These peaks were associated with the K lines of Mn, O, and C, which were observed at approximately 5.9 keV, 0.5 keV, and 0.26 keV, respectively. The elemental mapping confirmed that Mn and O were uniformly distributed throughout the samples. Quantitative analysis of the EDX data provided the elemental percentages, which enabled direct determination of the sample's composition. The elemental composition of the synthesized samples revealed a Mn : O ratio of 2 : 3 for M1, whereas P1M1, P2M1, and P3M1 consistently exhibited a ratio of 3 : 4. EDX analysis confirmed Mn_3_O_4_ as the main phase in the synthesized samples.^36^

#### XRD analysis

**Fig. 5:**
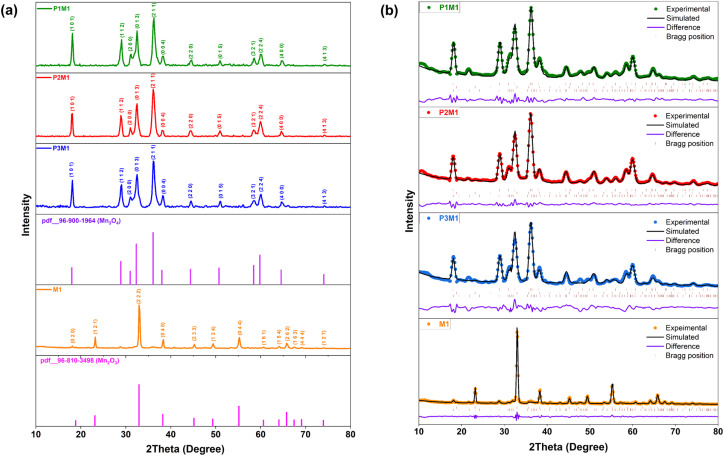
(a) Powder XRD pattern of the prepared samples, (b) Rietveld refinement plot of prepared samples displaying the simulated pattern, Bragg reflection positions, and the difference curve between the experimental and simulated data.

Phase identification and structural analysis of the synthesized powders were carried out using X-ray diffraction (XRD), and the corresponding diffraction patterns are presented in Fig. 5(a). The XRD pattern of the M1 sample, synthesized without PEG, predominantly corresponded to the cubic Mn_2_O_3_ phase (COD pdf #96-810-3498) with space group *Ia*-3 (206).^31^

In contrast, the PEG-assisted samples (P1M1, P2M1, and P3M1) mainly exhibited the tetragonal hausmannite Mn_3_O_4_ phase (COD pdf #96-900-1964) belonging to the *I*4_1_/*amd* (141) space group.^39^ Rietveld refinement further revealed the presence of minor secondary manganese oxide phases, particularly Mn_5_O_8_ (COD pdf #96-151-4101), in some samples, as illustrated in Fig. 5(b). The quantitative phase composition, along with the corresponding refinement parameters, is summarized in Table 1.

**Table 1:** Structural parameters obtained from Rietveld refinement of the synthesized manganese oxide samples, including phase composition, lattice parameters, crystallite size, microstrain, and refinement quality indicators

Sample	Major phase	Phase composition (%)			*a* (Å)	*c* (Å)	Crystallite size (nm)	Strain (%)	*R*_p_	*R*_wp_	GOF
		Mn_3_O_4_	Mn_2_O_3_	Mn_5_O_8_							
M1	Mn_2_O_3_ (cubic)	—	87.3	12.7	9.414	—	24.89	0.51	7.31	9.51	2.01
P1M1	Mn_3_O_4_ (tetragonal)	80.5	0.9	18.6	5.771	9.449	8.89	1.54	5.34	7.07	1.17
P2M1	Mn_3_O_4_ (tetragonal)	78.3	15.6	6.1	5.774	9.432	9.86	1.39	4.36	5.61	0.77
P3M1	Mn_3_O_4_ (tetragonal)	92.3	6.8	0.9	5.765	9.453	9.65	1.42	8.38	10.63	2.71

As confirmed by Rietveld refinement, template-free synthesis (M1) forms cubic Mn_2_O_3_. Conversely, PEG stabilizes the phase; increasing the PEG ratio from 1 : 0.5 (P1M1) to 1 : 2 (P3M1) systematically eliminates Mn_2_O_3_ to yield pure hausmannite Mn_3_O_4_, achieving a peak phase fraction of 92.3% in P3M1.

The lattice parameters obtained from the refinement were found to be in good agreement with standard crystallographic data. Slight variations in the lattice constants were observed among the PEG-assisted samples, which may be attributed to lattice distortion and defect formation during crystal growth. The crystallite sizes estimated from Rietveld refinement decreased significantly from 24.89 nm for M1 to approximately 9 nm for the PEG-assisted samples, indicating that PEG effectively restricted crystallite growth during synthesis. In contrast, the microstrain values increased for the PEG-containing samples, suggesting enhanced lattice distortion associated with the reduced crystallite size.

The refinement quality indicators, including *R*_p_, *R*_wp_, and GOF values, confirmed satisfactory agreement between the observed and calculated diffraction profiles, validating the structural model used for the analysis.

#### TEM analysis

**Fig. 6:**
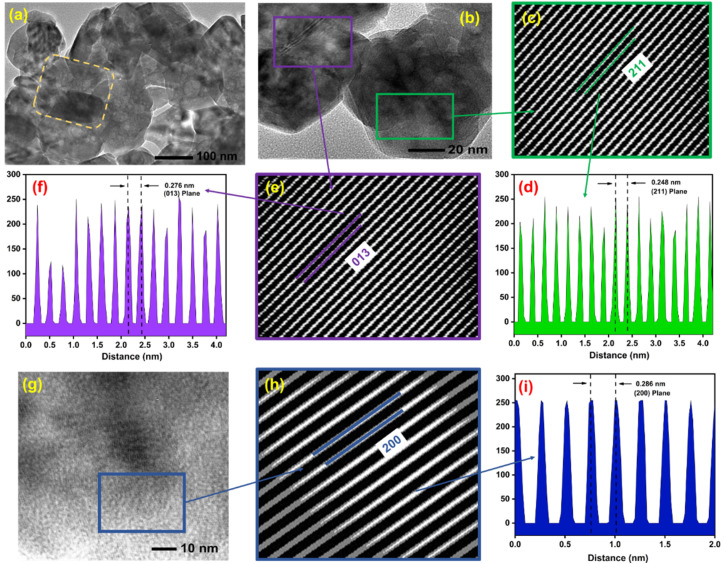
TEM image of the prepared P3M1 at different magnifications: scale bars of (a) 100 nm, (b) 20 nm, and (g) 10 nm. (c, e and h) FFT images of corresponding cross sections (b and g). (d, f and i) Line intensity plots of corresponding crystal planes from FFT images indicated by different colors.

TEM analysis was carried out to further support and validate the findings obtained from FESEM and XRD characterizations. The TEM micrographs of the synthesized P3M1 nanoparticles are presented in Fig. 6, where the particles appeared to exhibit spherical morphology with varying dimensions. A single well-defined particle was distinctly visible in Fig. 6(a), which was consistent with the morphological observations made from FESEM analysis. Well-resolved lattice fringes were identified in [Fig. 6(b) and (g)], confirming the high degree of crystallinity possessed by the Mn_3_O_4_ nanoparticles. Three cross-sectional regions from these two micrographs were selected and examined using ImageJ software. The corresponding FFT patterns shown in [Fig. 6(c), (e) and (h)], along with the line intensity profiles illustrated in [Fig. 6(d), (f) and (i)], enabled the identification of three distinct crystallographic planes, namely (211), (013), and (200), with their corresponding *d*-spacing values measured as 0.248, 0.276, and 0.286 nm, respectively. These experimentally determined *d*-spacing values were found to be in close agreement with those reported in the reference pattern COD pdf #96-900-1964, further confirming the crystalline phase of the prepared nanoparticles.^40^

### Solid state UV

**Fig. 7:**
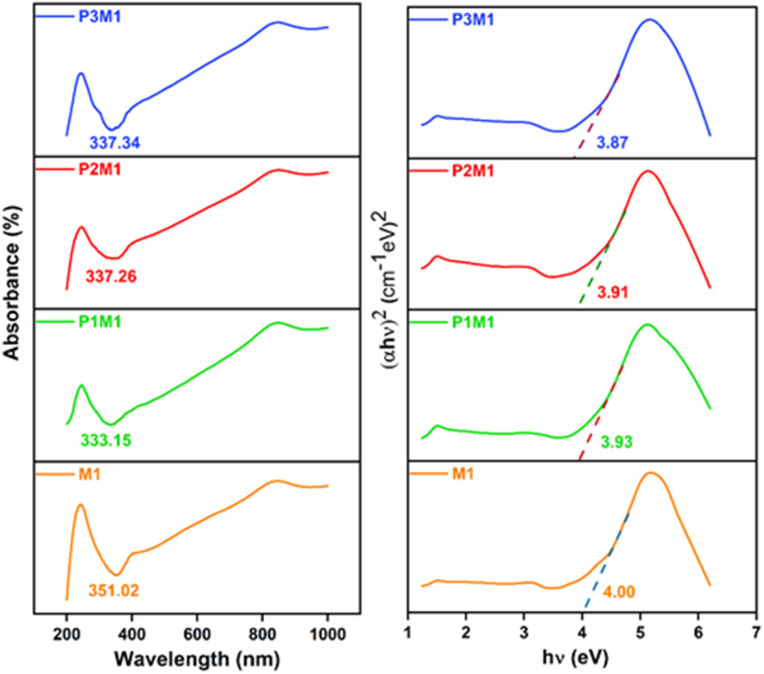
Optoelectronic properties of the prepared samples. (Left) UV-Vis absorption spectra. (Right) Absorption coefficient in the form of (*αhν*)^2^*vs. hν*.

The UV-Vis absorption spectra of the synthesized samples are presented in Fig. 7. The absorption coefficient (*α*) was determined using the equation^41^ -iv
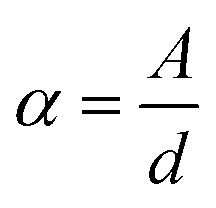
where, *d* denotes the specimen thickness. The photon dependency of the absorption coefficient was analyzed using Tauc's equation^42^ -

v (*αhν*)^2^ = *B*(*h − E*_g_)

where, *B* is the parameter that depends on the transition probability and *E*_g_ is the optical energy gap. The absorption coefficient, shown in Fig. 7, were obtained by plotting (*αhν*)^2^*versus hν*. The intercepts of the straight lines with the photon energy axis gave the optical band gap, which were found to be 4.00 eV, 3.93 eV, 3.91 eV and 3.87 eV for M1, P1M1, P2M1 and P3M1, respectively. This reduction in band gap energy could be attributed to the influence of PEG as a soft template during the synthesis process. By preventing aggregation and passivating surface traps, PEG may reduce mid-gap states, sharpen the conduction/valence bands, and effectively narrow the band gap. Since the optical band gap of semiconductors typically lies between >2.0 eV and <4.0 eV, these results confirmed the semiconducting nature of the prepared particles.

### Raman spectroscopy

**Fig. 8:**
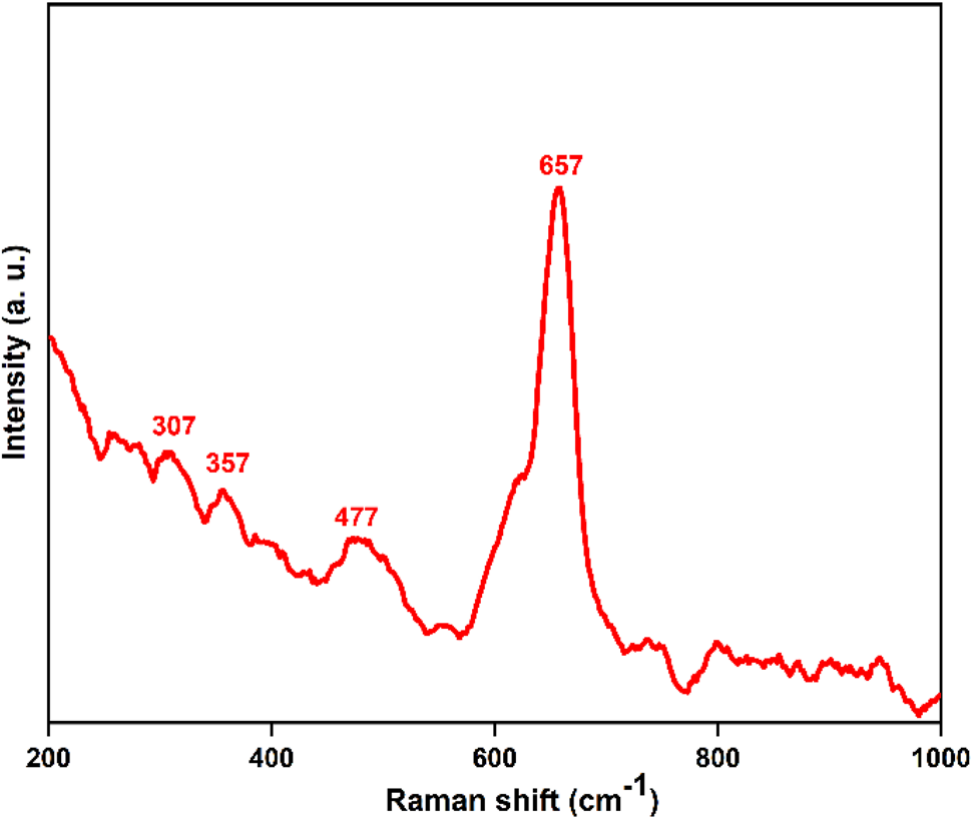
Raman spectrum of prepared P3M1.

The Raman spectrum of the synthesized P3M1 material (Fig. 8) showed four distinct peaks; a strong band at 657 cm^−1^ and three weaker, broader peaks at 307, 357, and 477 cm^−1^, which corresponded to the lattice vibrations of Mn_3_O_4_. The bands at 357 cm^−1^ and 477 cm^−1^ were attributed to the doubly degenerate T_2g_ symmetry mode, consistent with the tetragonal phase of Mn_3_O_4_.^43^ The A1g mode, linked to Mn–O breathing vibrations of Mn^2+^ ions in tetrahedral coordination, was found to have the strongest and sharpest peak at 657 cm^−1^. These results confirmed consistency between the synthesized material and commercially available and chemically generated Mn_3_O_4_ powders, as they were in good accord with the spectrum features of hausmannite.^44^

#### XPS analysis

**Fig. 9:**
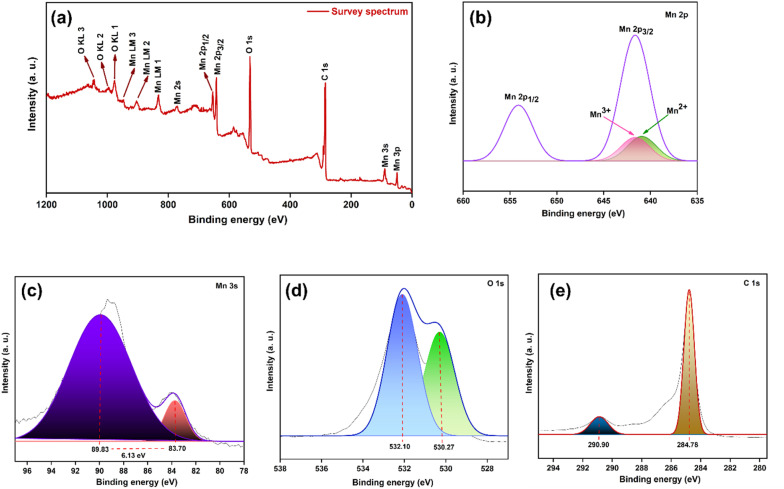
XPS spectra of P3M1 (a) complete survey spectrum. Core level XPS narrow spectra for (b) Mn 2p region; (c) Mn 3s region; (d) O 1s region, and (e) C 1s region. Gaussian deconvolution (fitted with the covered area).

XPS was employed to examine the elemental composition, electronic structure, and oxidation states of the synthesized P3M1. The survey and high-resolution spectra of the Mn_3_O_4_ nanomaterial are shown in [Fig. 9(a)]. The wide-scan survey spectrum confirmed the presence of Mn, O and C with well-defined signals corresponding to Mn 2p, Mn 3s, Mn 3p, O 1s, and C 1s, along with characteristic O KL and Mn LM Auger features. The high-resolution Mn 2p spectrum [Fig. 9(b)] exhibited two prominent peaks at binding energies of 654.17 and 642.18 eV, assigned to Mn 2p_1/2_ and Mn 2p_3/2_, respectively, with a spin–orbit splitting of ∼11.99 eV, consistent with the mixed-valence character of Mn_3_O_4_. The Mn 2p_3_/_2_ envelope was deconvoluted using Gaussian peak fitting, yielding two components at 641.66 and 640.85 eV, attributable to Mn^3+^ and Mn^2+^ species, respectively.^45^ Quantitative analysis of the fitted peak areas revealed that Mn^2+^ and Mn^3+^ account for 51.02% and 48.98% of the total surface manganese content, respectively, corresponding to a near-equimolar Mn^2+^ : Mn^3+^ ratio of approximately 1 : 1. This was further supported by the Mn 3s spectrum [Fig. 9(c)], which showed a characteristic multiplet splitting of ∼6.13 eV between the peaks at 89.83 and 83.70 eV consistent with mixed manganese oxidation states.^46^ The O 1s spectrum [Fig. 9(d)] was deconvoluted into two components located at 532.10 and 530.27 eV. The lower-energy peak was attributed to lattice oxygen (O^2−^), whereas the higher-energy was associated with surface hydroxyl species. These features corresponded to Mn–O–Mn bonding and Mn–OH surface groups, respectively, with the hydroxyl contribution originating from moisture adsorption upon environmental exposure.^47^ The C 1s spectrum [Fig. 9(e)] displayed a dominant peak at 284.78 eV arising from adventitious carbon, accompanied by a minor peak at 290.90 eV may be attributed to carbonate group. XPS analyses confirmed the successful formation of Mn_3_O_4_ with its characteristic mixed-valence Mn states and surface functionalities imparted by the PEG templating process.

### Wettability test

**Fig. 10:**
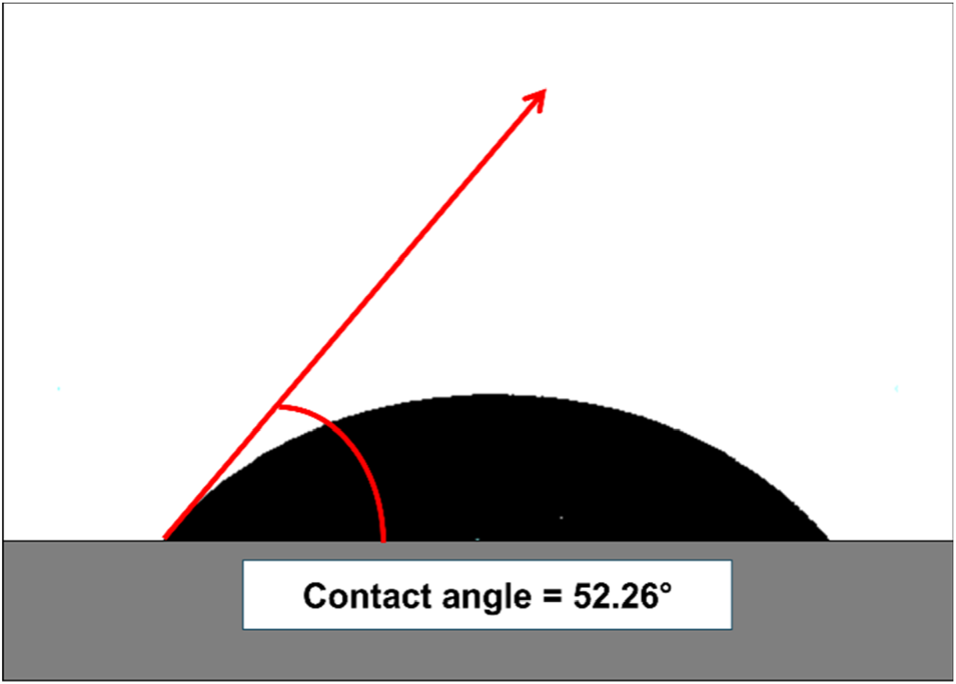
Water contact angle measurement of prepared P3M1.

Wettability measurements were carried out to examine the interaction between liquid and Mn_3_O_4_ powder. A smaller contact angle (*θ*) indicated higher wettability and a hydrophilic surface, whereas a larger contact angle (*θ*) corresponded to lower wettability and a hydrophobic nature. A contact angle of 0° represented complete wetting, while 180° denoted total non-wetting. Both super-hydrophilic and super-hydrophobic surfaces were considered important for various practical applications.^48^ As shown in Fig. 10, The water contact angle of the optimized P3M1 sample was measured to be 52.26°, indicating a hydrophilic surface. This relatively low contact angle could be attributed to the presence of surface hydroxyl groups (–OH), as confirmed by the broad O–H stretching band at ∼3425 cm^−1^ in FTIR and the hydroxyl component in the O 1s XPS spectrum. Comparable result was documented in earlier report.^49^ The incorporation of PEG during synthesis not only acted as a morphology-directing agent but also contributed to surface functionalization, which enhanced the surface polarity and wettability of the Mn_3_O_4_ nanoparticles.

Improved wettability plays a critical role in the electrochemical performance of aqueous rechargeable zinc-ion batteries. A hydrophilic cathode surface facilitates better penetration and spreading of the aqueous 2 M ZnSO_4_ electrolyte within the porous electrode structure. This leads to enhanced electrode–electrolyte interfacial contact, reduced charge transfer resistance, shorter diffusion pathways for hydrated Zn^2+^ and H^+^ ions, increased utilization of active redox sites. These factors collectively contribute to the observed reversible CV behavior, faster Zn^2+^ (de)intercalation kinetics, and the high specific discharge capacity of 274.82 mA h g^−1^ at 0.1 A g^−1^. The progressive increase in capacity during the initial ∼40 cycles can also be partly attributed to the gradual improvement in electrolyte wetting and activation of previously inaccessible sites within the nanostructured Mn_3_O_4_. Furthermore, the hydrophilic nature helps mitigate volume expansion stress during repeated cycling by promoting uniform ion distribution, which partially accounts for the respectable capacity retention of 76.12% after 500 cycles. This observation is consistent with recent findings in manganese-based cathodes for ARZIBs. Yang *et al.* (2025) constructed a TiO_2_ interphase on δ-MnO_2_*via* chemical liquid-phase deposition, which simultaneously suppressed manganese dissolution and improved electrode wettability. The enhanced ion transport at the interface resulted in excellent rate capability and cycling stability, delivering a specific capacity of 310 mA h g^−1^ at 0.2 A g^−1^ and 87.6% capacity retention after 1000 cycles at 10 A g^−1^.^50^ Similarly, Zhang *et al.* (2020) systematically investigated the effect of cathode wettability in AZIBs using cellulose nanowhiskers/graphene/MnO_2_ composites with contact angles ranging from 64.70° to 115.85°. They demonstrated a parabolic relationship between wettability and performance, with the optimal performance (384 mA h g^−1^ at 1C and ultra-long cycling over 5000 cycles at 20C) achieved at a moderately hydrophobic contact angle of ∼103°.^51^

### Electrochemical analysis

#### CV assessment

**Fig. 11:**
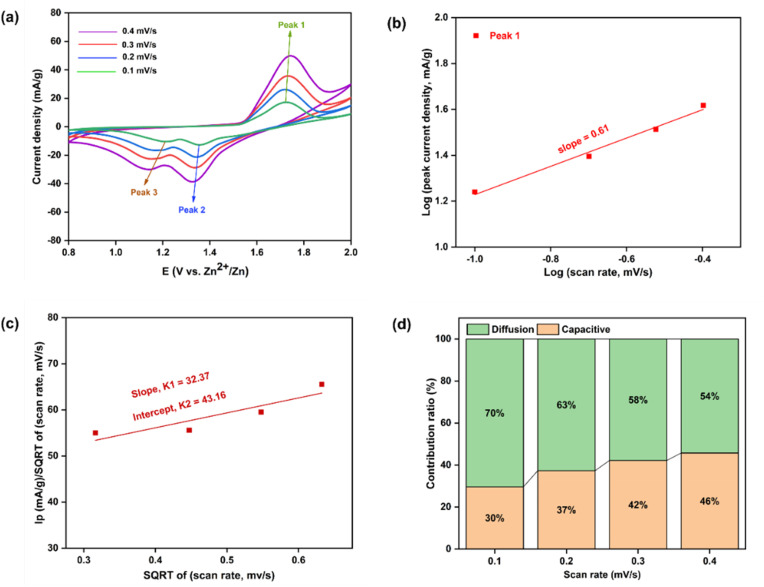
CV curves of P3M1-manufactured coin cell (With template) (a) at 0.1, 0.2, 0.3 and 0.4 mV s^−1^ scan rates, (b) the fitted lines: log(peak current density) *vs.* log(scan rate), (c) SQRT of (scan rate) *vs. I*_p_/SQRT of (scan rate) with a fitted line (d) percentages of capacitive and diffusion contribution at different scan rates.

Within the electrochemical window of +0.8 V to +2.0 V, CV measurements were performed for the fabricated P3M1 coin cell at various scan rates (0.1, 0.2, 0.3, and 0.4 mV s^−1^). In Fig. 11(a), a clear anodic peak was observed from +1.53 to +1.83 V, complemented by two cathodic peaks at +1.41 to +1.24 V and +1.19 to +1.05 V. Similar oxidation and reduction peaks were detected in each CV profile across the different scan rates. The anodic scan revealed a sharp oxidation peak at +1.74 V, signifying the phase transformation of Mn_3_O_4_ to Mn_5_O_8_ as Zn^2+^ ions were stored within the interlayer. On the reverse scan, the two reduction peaks corresponded to the release of Zn^2+^ ions and the extraction of H^+^ ion – the latter occurring alongside the reduction of Mn(ii) to Mn(iii) – thereby confirming a simultaneous H^+^ and Zn^2+^ co-deintercalation mechanism.

The peak heights increased with faster scan rates, and the anodic peaks shifted towards the right while cathode peaks shifted to the left, demonstrating the possible slight adsorption of Zn^2+^ ions onto the cathode surface responsible for Zn^2+^ ion storage.^52^ The probable electrochemical reactions were expressed as follows:^32^Anode reaction: Zn ↔ Zn^2+^ + 2e^−^Cathode reaction: 2Mn_3_O_4_ + 2e^−^ ↔ Mn_5_O_8_ + Mn^2+^H_2_O ↔ H^+^ + OH^−^

An assessment of the CV behavior was carried out using Dunn's approach^53^ -

vi Log *i* = Log *a* + *b* Log *v*

where *a* and *b* are constants. The slope value (*b*) was obtained between 0.5 and 1.0. A slope of 0.5 indicated diffusion-controlled behavior, while 1.0 denoted capacitive behavior. The anodic peak (Peak 1) exhibited a slope of 0.6 [Fig. 11(b)], implying that the dominant charge storage mechanism was diffusion-controlled pseudo capacitance associated with battery-type behavior.^54^ The presence of Mn_3_O_4_ nanoparticles enhanced Zn^2+^ storage by reducing diffusion limitations. The capacitive contribution ratio was calculated based on the anodic response using the following relation:^55^i_p_(*v*) = *k*_1_*v* + *k*_2_*v*^1/2^vii
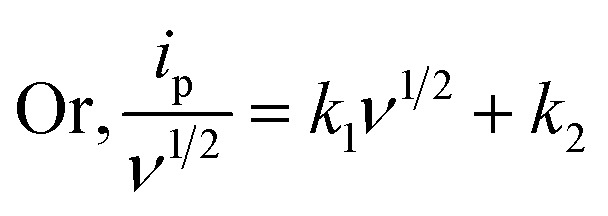
where, *k*_1_ and *k*_2_ are the slope and intercept of *ν*^1/2^*vs.*
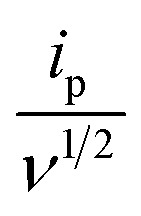
 plot [Fig. 11(c)]. The pseudo-charge storage contribution was represented by *k*_1_*ν*, while the insertion type capacity was represented by *k*_2_*ν*^1/2^.^56^ At 0.4 mV s^−1^, the capacitive contribution was calculated to be around 46%. With increasing scan rates of 0.1, 0.2, 0.3, and 0.4 mV s^−1^, the capacitive contributions were 30%, 37%, 42%, and 46%, respectively. The capacitive contribution progressively rose with scan rate, as seen in [Fig. 11(d)], reaching a maximum of 46% at 0.4 mV s^−1^. Higher scan rates may yield higher capacitive coverage. Compared to the similar values reported in previous studies, these results were lower. Crystallinity could be used to explain this. Although the crystallite size was small, it offered relatively few active sites for deintercalation-driven energy storage.^31^ Therefore, at high current densities, the overall storage process was predominantly capacitance-controlled.

**Fig. 12:**
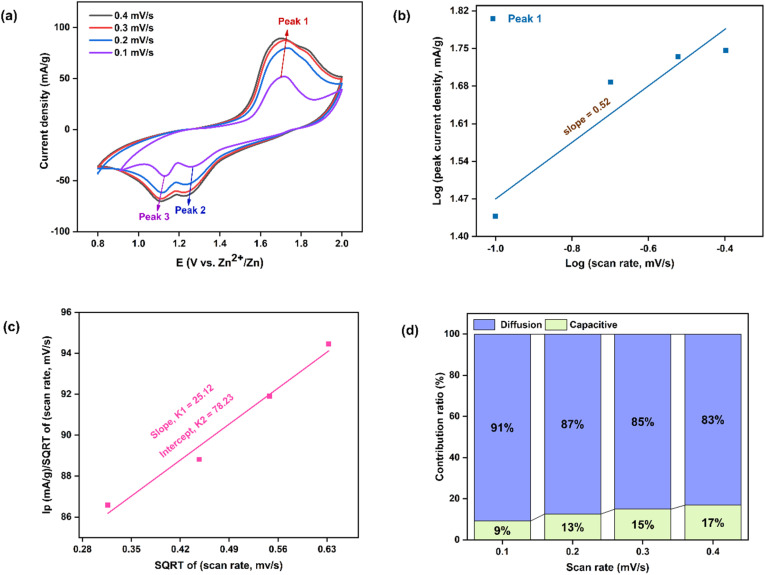
CV curves of M1-manufactured coin cell (Without template) (a) at 0.1, 0.2, 0.3 and 0.4 mV s^−1^ scan rates, (b) the fitted lines: log(peak current density) *vs.* log(scan rate), (c) SQRT of (scan rate) *vs. I*_p_/SQRT of (scan rate) with a fitted line (d) percentages of capacitive and diffusion contribution at different scan rates.

For the M1 cathode-based cell, a distinct anodic peak was observed in the potential range of +1.54 to +1.86 V, along with two cathodic peaks located between +1.36 to +1.19 V and +1.19 to +1.03 V [Fig. 12(a)]. Well-defined and consistent redox peaks were maintained at different scan rates. The calculated *b*-value for the anodic peak was 0.52 [Fig. 12(b)], suggesting that the charge storage process was predominantly diffusion-controlled. Furthermore, the capacitive contribution derived from the anodic response reached only ∼17% at 0.4 mV s^−1^ [Fig. 12(d)], which was significantly lower than that of the P3M1-based cathode.

### BCD analysis

**Fig. 13:**
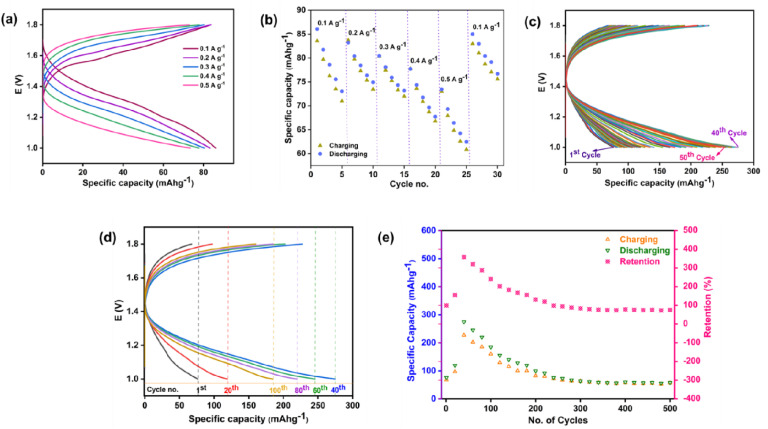
BCD profile of the assembled P3M1 coin cell: (a) specific capacity at different current density, (b) rate capability at different current density, (c) specific capacity of initial 50 cycles at 0.1 A g^−1^ current density, (d) specific capacity at 0.1 A g^−1^ current density, (e) cycling behavior, specific capacity and specific discharge capacity retention at 0.1 A g^−1^ over 500 cycles.

Fig. 13(a) illustrates BCD profile of the fabricated coin cell employing P3M1 as the cathode material, evaluated across a range of applied current densities. The results demonstrated that as the current density was progressively increased from 0.1 to 0.5 A g^−1^, the specific discharge capacity exhibited a consistent declining trend, yielding values of 86.05, 83.21, 80.43, 77.72, and 73.44 mAh g^−1^, respectively. The rate capability performance of the fabricated cell was subsequently evaluated at the same current densities, as depicted in Fig. 13(b). Upon returning the current density to its original value of 0.1 A g^−1^, the specific discharge capacity was recovered to 84.97 mAh g^−1^, which closely approached the initial capacity of 86.05 mAh g^−1^. This corresponded to a capacity retention of approximately 98.74%. Fig. 13(c) depicted the charge–discharge curves recorded over the first 50 cycles.

Charging-discharging was done at applied current density 0.1 A g^−1^ for 500 cycles. The initial specific discharge capacity was 76.47 mAh g^−1^, which increased to 274.82 mAh g^−1^ as the cycling proceeded up to 40th cycles, then decreased gradually for the rest of the cycling, and reached 58.21 mAh g^−1^ at 500th cycle [Fig. 13(d) and (e)]. The crystal structure of Mn_3_O_4_ is a combination of mixed valence- Mn^2+^ in tetrahedral sites and Mn^3+^ in octahedral sites. The coexistence of Mn^2+^ and Mn^3+^ allows multiple redox couples. But the structure of Mn_3_O_4_ is not flexible like MnO_2_, thus restricted for Zn^2+^ ion diffusion at the initial charging-discharging stages. When the battery discharges, Zn^2+^ ions from the electrolyte (ZnSO_4_) migrated to the cathode. Instead of just intercalation, they trigger conversion-like reactions.^57,58^

viii Mn_3_O_4_ + *x*Zn^2+^ + 2*x*e^−^ → MnO_2_/MnOOH + Mn_(aq)_^2+^

Some Mn^3+^ is reduced to Mn^2+^, dissolves into the electrolyte, while part of solid transforms into MnO_2_/MnOOH nanosheets, which is a more open structure than initial Mn_3_O_4_, responsible for higher capacity. While intercalation, Zn^2+^ ion along with H^+^ ion enter to the cathode from the electrolyte starting water dissociation. The water molecules solvate the Zn^2+^ ion. A part of the hydration shell of the solvated ion retains in the cathode, helping stabilize its structure after intercalation. Some part of the cathode experiences internal cracking, resulting in the exposing of new sites. This formation and stabilization of MnO_2_/MnOOH in the cathode surface/interior lead to the activation of the cathode and lead its gradual capacity rising. In this case, up to 40 cycles, capacity increased, and then gradually decreased for rest of the 500th cycles. From 1st to 40th cycles, the restructuring and stabilization of cathode led to the co-insertion of Zn^2+^ and H^+^ ions, fluctuation between Mn^3+^/Mn^4+^ and extra H^+^ ion insertion for charge balance, resulting layered/amorphous like cathode (MnO_2_/MnOOH) for more diffusion channels, improving the conductivity as well as the capacity for mixed valency states.^59^ After 40th cycle, the cathode got stable, no cracking of the cathode interior happened then, continuous dissolving of Mn^2+^ ion was lost. The hydrated Zn^2+^ radius developed strong strain to the cathode crystal lattice. Thus repeated (de)intercalation cracked the structure externally, leading collapse of stabilized cathode. Sometimes by-products are formed, which deposited at the electrode surface, blocking the pores, hindered the Zn^2+^ ion transport, leading increased resistance. Thus, after 40 cycles, due to side reaction with electrolyte, deposition of by-product on the cathode surface, accumulation of intermediate product between Zn^2+^ ion and cathode, the active mass of the cathode decreased, resulting capacity fading to 500th cycling.^33^ It was also noticed that the discharge capacity was slightly higher than the charging capacity. The coulombic efficiency was more than 100%, it was higher at the beginning of the cycling and at the cycling proceeded its value decreased and catch near to 100% (Table 2). The coulombic efficiency exceeding 100% suggested the occurrence of additional discharge–side reactions, which might have included partial Mn-related redox contributions. This behavior indicated that the Zn-ion insertion mechanism was not purely governed by Zn-ion storage, but rather involved a combined contribution from Zn-ion intercalation and Mn-redox-associated pseudocapacitive processes. As observed in Fig. 13(d), the specific capacity initially increased during the early cycling stages and subsequently decreased gradually from the 40th to the 500th cycle. The initial increase in capacity was attributed to the electrochemical activation of the electrode material. This phenomenon was likely associated with the coexistence of mixed Mn oxidation states (Mn^2+^/Mn^3+^) within the cathode, which enabled multiple redox couples and contributed to enhanced electrochemical activity during the initial cycles. During charging, Zn^2+^ intercalates into the cathode. At the same time, some of the Mn from the cathode dissolves into the electrolyte as Mn^2+^ due to disproportionation of Mn^3+^.^60^

ix 2Mn^3+^ → Mn^2+^ + Mn^4+^

**Table 2:** Charging capacity, discharging capacity, and coulombic efficiency values at different cycle numbers

Cycle no.	Charging capacity	Discharging capacity	Coulombic efficiency
1	67.77	76.47394	112.83139
20	97.49	119.18713	122.24734
40	227.36	274.82528	120.87407
60	202.37	245.50811	121.31107
80	185.74	219.85775	118.36401
100	159.94	184.83362	115.56412
120	129.85	155.07914	119.4287
140	129.85	140.28341	108.0343
160	100.53	128.26965	127.58235
180	100.71	119.66947	118.82294
200	82.19	99.97701	121.62908
220	80.70	92.42089	114.5214
240	80.70	75.89144	94.03928
260	80.70	73.04957	90.51784
280	64.46	67.40698	104.56057
300	63.37	64.03091	101.02972
320	59.73	61.30051	102.62816
340	58.43	58.94126	100.86943
360	56.88	57.55666	101.18092
380	55.09	56.88027	103.23998
400	57.25	60.29231	105.29897
420	54.81	58.65657	107.00749
440	54.86	57.45062	104.71574
460	55.68	58.42664	104.93186
480	53.03	56.20071	105.97266
500	56.61	58.21741	102.83312

While discharging, only a part of inserted Zn^2+^ is extracted, thus lowering of capacity occurs. But Mn^2+^ from the electrolytes can chemically or electrochemically redeposit as MnO_2_/MnOOH or Mn_2_O_3_/MnOOH on the cathode surface. This side reaction adds extra capacity on discharge due to the participation of more electron than expected per unit of cathode. Thus, though the discharge is not fully reversed but the capacity is higher for extra e^−^ involvement. Another reason could be the electrolyte side reaction, when H^+^ is intercalated to the cathode with Zn^2+^ from the electrolyte, due to the solvation ability of Zn^2+^ with the water content present within the cell. Upon discharging the release of H^+^ contributes additional discharge capacity.^61^ Besides, the phase transformation up to 40th cycles, allowed more Zn^2+^/H^+^ insertion/excretion sites than were accessible at the beginning, resulting higher coulombic efficiency and specific discharge capacity retention. After 300, 400 and 500 cycles, the specific discharge capacity retention were found to be 83.72%, 78.84% and 76.12%, respectively. Although Mn-oxide-based cathodes in ARZIBs generally exhibit voltage plateaus over a relatively broad potential range (∼+0.8 to +2.0 V), the electrochemical measurements in this study were intentionally restricted to a narrower operating window of +1.0 to +1.8 V. Within this selected voltage range, the BCD profiles predominantly exhibited sloping characteristics rather than well-defined voltage plateaus. It was observed that extending the potential window beyond this range resulted in poorer capacity retention. Therefore, the electrochemical investigations were conducted within the optimized voltage range while varying the applied current densities under a fixed voltage window [Fig. 13(a) and (b)]. The restricted operating range was deliberately chosen to minimize irreversible side reactions and suppress possible parasitic secondary reactions, thereby improving the electrochemical stability of the system.

**Fig. 14:**
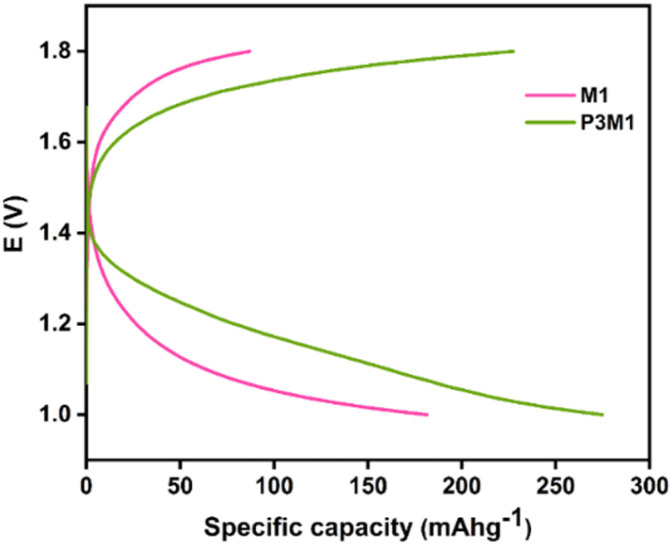
Comparative battery charge–discharge curves of M1 and P3M1 cathodes.

Fig. 14 compares the BCD profiles of the M1 and P3M1 cathode-based coin cells. The M1 cathode delivered a specific discharge capacity of 181.44 mAh g^−1^, whereas the P3M1 cathode achieved a significantly higher value of 274.82 mAh g^−1^ at 0.1 A g^−1^ -representing a capacity improvement of approximately 51.5%. This substantial gain was attributed to the role of the PEG template in directing structurally ordered morphology during synthesis, which facilitated more accessible active sites for ion intercalation/deintercalation.

#### EIS analysis

**Fig. 15:**
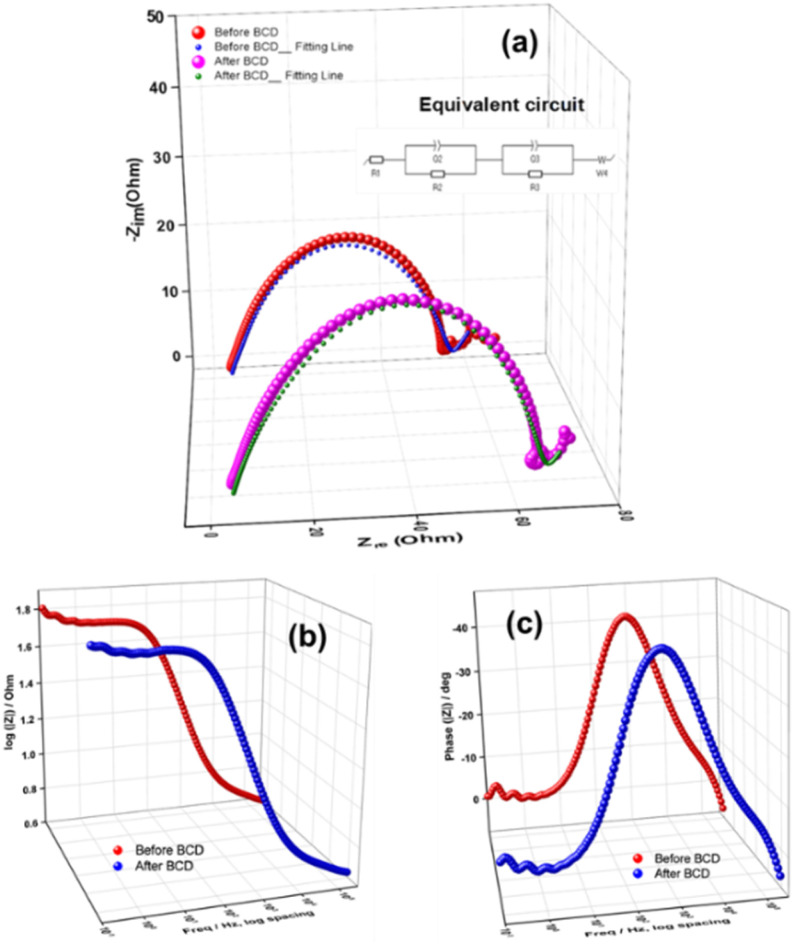
EIS of fabricated P3M1 battery using prepared material: (a) Nyquist plot, inside unveiling fitting circuit, Bode plots of frequency-dependent (b) impedances, and (c) phase angles.

[Fig. 15(a)] illustrates the Nyquist plots of the fabricated P3M1 coin cell before and after BCD of 500 cycles. The experiment was conducted at +1.8 V over a frequency range spanning from 300 kHz to 100 mHz. To analyze the Nyquist response, the equivalent circuit model *R*_1_ + *Q*_2_/*R*_2_ + *Q*_3_/*R*_3_ + *W*_4_ was applied. In this model, *R*_1_ corresponded to the solution resistance (*R*_s_), *R*_2_ represented the resistance at the electrode–electrolyte interface due to the SEI layer (*R*_f_), *R*_3_ denoted the charge transfer resistance (*R*_ct_), *Q*_2_ and *Q*_3_ were constant phase elements (CPEs) associated with porous structures, and *W*_4_ was the Warburg impedance (*W*_s_).^62^ For the fresh P3M1 cell, the extracted values were *R*_s_ = 4.178 Ω, *R*_f_ = 11.56 Ω, *R*_ct_ = 32.05 Ω, and *W*_s_ = 2.324 Ω s^−1/2^. After 500 BCD cycles, the values increased to *R*_s_ = 4.44 Ω, *R*_f_ = 27.94 Ω, *R*_ct_ = 33.71 Ω, and *W*_s_ = 2.573 Ω s^−1/2^. These outcomes indicated that resistances were lower in the fresh cell but rose after BCD cycling. The equivalent-circuit model provided a satisfactory fit at higher frequencies but deviated at lower frequencies, which was attributed to the development of secondary capacitive effects within the cell. The coin cell had been fabricated manually; cathode material was pasted onto the current collector foil and ordinary filter paper was used as the separator. It was possible that high-pressure crimping caused some cathode material to infiltrate the separator. Moreover, during prolonged BCD cycling, portions of the cathode likely detached, migrated through the filter paper, and accumulated on or formed a layer over the anode surface. These phenomena presumably contributed to reduced discharge capacity retention and reflected non-uniform mass transport within the cell.^32^ [Fig. 15(b)]. depicted the Bode plots of impedance *versus* frequency. Impedance remained low at high frequencies but increased as frequency decreased, confirming the capacitive energy-storage behavior of Mn_3_O_4_ nanoparticles [Fig. 15(c)]. displayed the phase-angle plots; the maximum phase angle increased slightly after BCD of 500 cycles indicating pseudocapacitive Zn^2+^ ion storage at low frequencies.

### Post mortem analysis

**Fig. 16:**
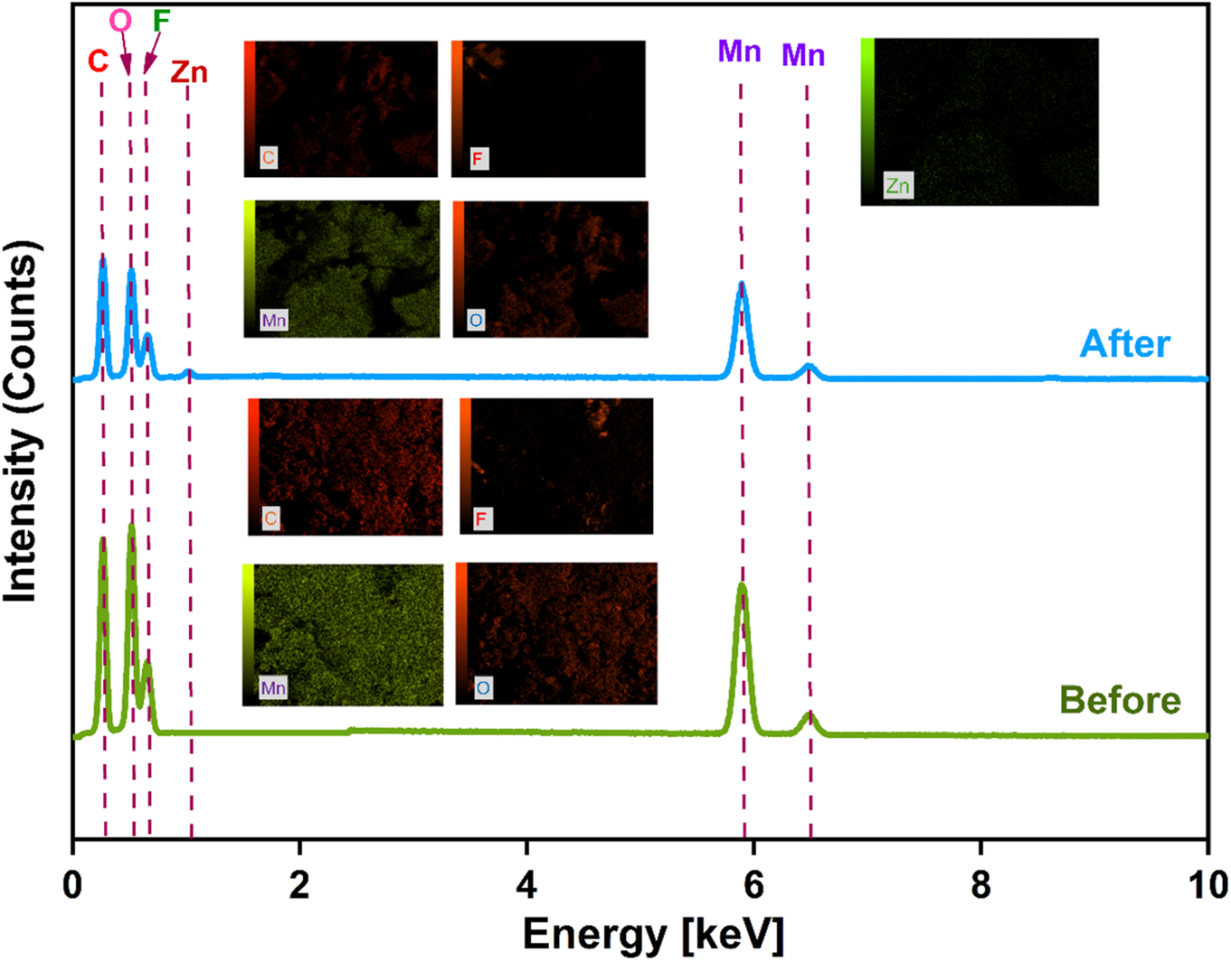
EDX spectra of cathodes before and after BCD (inside showing elemental mapping).

To investigate the reaction mechanism occurring inside the coin cell, post-mortem characterization of the cathode material was carried out. After completing the BCD test, the coin cell was carefully dismantled using a sharp needle and hammer. The cathode material was collected, rinsed with DI water, and subsequently analyzed by EDX. The EDX spectra of the fresh cathode (before BCD) and the used cathode (after BCD) are presented in Fig. 16. Both cathodes exhibited Mn and O signals corresponding to Mn_3_O_4_, along with a small amount of F originating from the PVDF binder. Compared to the fresh cathode, the used cathode showed the presence of Zn, indicating Zn^2+^ ion insertion into the Mn_3_O_4_ structure during cycling. In addition, the Mn and O contents in the used cathode decreased by 0.12% and 2.91%, respectively, relative to the fresh cathode, which may be attributed to side reactions and the partial dissolution of Mn_3_O_4_ during the repeated charge–discharge process. This observation may be associated with the decline in capacity retention after prolonged BCD cycling. The formation of intermediate compounds due to Zn^2+^ (de)intercalation could make the electrochemical reaction partially irreversible, leading to the formation of Zn_*x*_Mn_2_O_3_ species within the cathode and contributing to the reduced cycling stability after 500 cycles. The specific discharge capacities with respect to applied current densities of different Mn_3_O_4_ based materials are listed in Table 3. Considering the straightforward synthesis route and the low cost associated with cathode fabrication, the present work demonstrates a simple and economically viable approach for developing competitive cathode materials for ARZIBs.

**Table 3:** List of specific discharge capacities with respect to applied current densities of Mn_3_O_4_ based cathodes in ARZIBs

Cathode materials	Applied current density (A g^−1^)	Specific discharge capacity (mAh g^−1^)	References
Mn_3_O_4_	0.1	221	58
Mn_3_O_4_	2	87	63
Mn_3_O_4_	0.1	239.2	21
MOF-derived Mn_3_O_4_	3	124.3	52
Mn_3_O_4_@C	0.5	209.6	64
Mn_3_O_4_/GO composite	0.1	215.6	65
Core-shell Mn_3_O_4_/carbon (Mn_3_O_4_@C) fiber	0.3	215.8	66
Mn_3_O_4_ @N dopped carbon coated carbon cloth	0.2	265.8	67
Mn_3_O_4_ @N dopped carbon matrix composite nanorods	0.1	280	68
Mn_3_O_4_	0.1	280.9	69
Copper-ion-doped Mn_3_O_4_	0.1	360	70
Zinc-doped Mn_3_O_4_	0.5	116	71
Cerium-modified Mn_3_O_4_	0.5	156.36	72
Mn_3_O_4_	1	120	73
Mn_3_O_4_/MnCO_3_	3	91.3	74
Co-doped Mn_3_O_4_	0.2	237	75
Mn_3_O_4_	5	123	76
Mn_3_O_4_	0.1	120	77
Mn_3_O_4_	0.1	219.33	31
Mn_3_O_4_	0.1	240.75	32
**Mn**_**3**_ **O**_**4**_	**0.1**	**274.82**	**This work**

## Conclusion

Mn_3_O_4_ nanoparticle of tuned morphology was synthesized through a facile one-step calcination of manganese(ii) acetate tetrahydrate employing polyethylene glycol 3350 (PEG) as a soft template. The prepared Mn_3_O_4_ showed semiconducting properties and was employed as the cathode in CR-2032 coin-cell ARZIBs. The CV analysis demonstrated that the cell exhibited a reversible process driven by diffusion-controlled ion insertion and extraction between the anode and cathode. At a current density of 0.1 A g^−1^, the assembled battery delivered an initial discharge capacity of 274.82 mAh g^−1^ at 40th cycle and maintained 76.12% of its capacity after 500 charge–discharge cycles.

## Conflicts of interest

There are no conflicts of interest to declare.

## Data Availability

Data will be made available on request.

## References

[cit1] Hassan M. Z., Sheikh M. S. I., Ahamed P., Yousuf M. A. (2025). Carbohydr. Polym. Technol. Appl..

[cit2] Goodenough J. B., Kim Y. (2010). Chem. Mater..

[cit3] Zhang H., Liu T., Li K., Liu Y., Hu J., Zuo Q., Jiang L. (2026). J. Energy Storage.

[cit4] Meng J., Yang Z., Chen L., Qin H., Cui F., Jiang Y., Zeng X. (2020). Mater. Today Energy.

[cit5] Zhang H., Meng Y., Wang F., Zhang Z., Yang Z. (2026). AIChE J..

[cit6] Hao W. , Guo F., Li J. and Xie J., Influence of Physical Parameters on Lithium Dendrite Growth Based on Phase Field Theory Metals, 2026, p. 41

[cit7] Jiang M., Zhang H., Wang F., Zhang Z., Yang Z. (2025). Chem. Eng. Sci..

[cit8] Wang F., Borodin O., Gao T., Fan X., Sun W., Han F., Faraone A., Dura J. A., Xu K., Wang C. (2018). Nat. Mater..

[cit9] Yang X., Xie Y., Zheng X., Gai J., Zhao Y., Zhang Q., Yan H. (2026). Ceram. Int..

[cit10] ed. M. M. Islam, M. S. I. Sheikh, M. A. B. H. Susan, M. M. Islam, R. K. Gupta, Springer International Publishing, Cham, 2022, pp. , pp. 265–287

[cit11] Hao J., Zhang S., Wu H., Yuan L., Davey K., Qiao S.-Z. (2024). Chem. Soc. Rev..

[cit12] Li X., Jin T., Wang Z., Chen Y., Yun C., Shang J., Ge Y., Lu T., Zhuang W., Ma Y., Qi Z. (2026). Adv. Sci..

[cit13] Hashem Abdelmohsen A., El-Khodary S. A., Ismail N., Song Z., Lian J. (2025). Chem.–Eur. J..

[cit14] Ruan P., Liang S., Lu B., Fan H. J., Zhou J. (2022). Angew. Chem..

[cit15] Guo J., Gao Y., Cao X., Li L., Yu X., Chi S., Liu H., Tian G., Zhao X. (2025). Renewable Energy.

[cit16] Zhou A., Chi R., Shi Y., Zhao X., Li X., Kou Z., Zhang Z., Zhang X., Nie G. (2023). Mater. Today Chem..

[cit17] Guo Y., Zhang Y., Lu H. (2022). Battery Energy.

[cit18] Zhao S., Wang J., Wu F., Chen X., Wu F., Zou J., Zhou H., Xie F., Wang W., Gao P., Fan W., Ye W. (2026). Appl. Catal., B.

[cit19] Wang Y., Wang Y., Tang X., Zhang L., Xiao D., Zhang X., Zhao Q. (2025). Appl. Surf. Sci..

[cit20] Zhao Y. (2022). J. Mater. Res. Technol..

[cit21] Hao J., Mou J., Zhang J., Dong L., Liu W., Xu C., Kang F. (2018). Electrochim. Acta.

[cit22] Liu A., Wu F., Zhang Y., Zhou J., Zhou Y., Xie M. (2022). Small.

[cit23] Madan C. , Kumari S., Halder A., Kumar V., Ayoub I., Sharma V. and Swart H. C., in Optical Properties of Metal Oxide Nanostructures, Springer Nature Singapore, Singapore, 2023, pp. 291–330

[cit24] He B., Zhang H., Lu J., Yang T., Yan S., Li Z., Han X., Cai S. (2026). J. Alloys Compd..

[cit25] Ren Q., Yuan Y., Wang S. (2022). ACS Appl. Mater. Interfaces.

[cit26] Shameli K., Bin Ahmad M., Jazayeri S. D., Sedaghat S., Shabanzadeh P., Jahangirian H., Mahdavi M., Abdollahi Y. (2012). Int. J. Mol. Sci..

[cit27] Hong S., Jin S., Deng Y., Garcia-Mendez R., Kim K.-I., Utomo N., Archer L. A. (2023). ACS Energy Lett..

[cit28] Li Y., Zhu G., Qin M., Zhou Y., Zhao M., Shen Y., Zhao H. (2021). J. Porous Mater..

[cit29] Chen M., Xiang X., Chen D., Liao Y., Huang Q., Li W. (2015). J. Power Sources.

[cit30] Zhou J., Shan L., Wu Z., Guo X., Fang G., Liang S. (2018). Chem. Commun..

[cit31] Tonu N. T., Ahamed P., Yousuf M. A. (2024). Ionics.

[cit32] Tonu N. T., Ahamed P., Yousuf M. A. (2024). PloS One.

[cit33] Tonu N. T., Yousuf M. A., Ahamed P., Hasan M. M. (2025). RSC Adv..

[cit34] Li X., Mao J., Cheng F. (2024). ACS Appl. Energy Mater..

[cit35] Hosny N. M., Dahshan A. (2012). Mater. Chem. Phys..

[cit36] Atique Ullah A. K. M., Fazle Kibria A. K. M., Akter M., Khan M. N. I., Tareq A. R. M., Firoz S. H. (2017). Water Conserv. Sci. Eng..

[cit37] Yousefi T., Golikand A. N., Mashhadizadeh M. H., Aghazadeh M. (2012). Curr. Appl. Phys..

[cit38] Mahajan H., Kumar S., Sharma A., Mohammed I., Thakur M., Kaur A., Srivastava A. K. (2023). J. Sol-Gel Sci. Technol..

[cit39] Satomi K. I. (1961). J. Phys. Soc. Jpn..

[cit40] Zhang P., Zhan Y., Cai B., Hao C., Wang J., Liu C., Meng Z., Yin Z., Chen Q. (2010). Nano Res..

[cit41] Zhong M., Jang M. (2011). Atmos. Environ..

[cit42] López R., Gómez R. (2012). J. Sol-Gel Sci. Technol..

[cit43] Shaik D. P. M. D., Rosaiah P., Hussain O. M. (2016). Mater. Today: Proc..

[cit44] Shah H. U., Wang F., Toufiq A. M., Ali S., Khan Z. U. H., Li Y., Hu J., He K. (2018). J. Nanosci. Nanotechnol..

[cit45] Barreto J., Bagus P. S., Stavale F. (2025). J. Phys.: Condens. Matter.

[cit46] Moses Ezhil Raj A., Victoria S. G., Jothy V. B., Ravidhas C., Wollschläger J., Suendorf M., Neumann M., Jayachandran M., Sanjeeviraja C. (2010). Appl. Surf. Sci..

[cit47] Zhang J., Chu R., Chen Y., Zeng Y., Zhang Y., Guo H. (2019). Electrochim. Acta.

[cit48] Sun R.-D., Nakajima A., Fujishima A., Watanabe T., Hashimoto K. (2001). J. Phys. Chem. B.

[cit49] Dubal D. P., Dhawale D. S., Salunkhe R. R., Pawar S. M., Lokhande C. D. (2010). Appl. Surf. Sci..

[cit50] Yang T., Zuo Y., Yan S., Zhang H., Zhang H., Ling L., He B., Lu J., Cai S. (2025). Surf. Interfac..

[cit51] Zhang X., Li J., Ao H., Liu D., Shi L., Wang C., Zhu Y., Qian Y. (2020). Energy Storage Mater..

[cit52] Yin C., Chen J., Pan C.-L., Pan Y., Hu J. (2022). ACS Appl. Energy Mater..

[cit53] Liu X., Cui L., Yu K., Lv J., Liu Y., Ma Y., Zhou B. (2021). Inorg. Chem..

[cit54] Muller G. A., Cook J. B., Kim H.-S., Tolbert S. H., Dunn B. (2015). Nano Lett..

[cit55] Lan K., Wei Q., Wang R., Xia Y., Tan S., Wang Y., Elzatahry A., Feng P., Mai L., Zhao D. (2019). J. Am. Chem. Soc..

[cit56] Chao D., Liang P., Chen Z., Bai L., Shen H., Liu X., Xia X., Zhao Y., Savilov S. V., Lin J., Shen Z. X. (2016). ACS Nano.

[cit57] Zhang R., Ma Q., Chen S., Huang J., Zhu L., Liu J. (2020). Solid State Ionics.

[cit58] Wang L., Cao X., Xu L., Chen J., Zheng J. (2018). ACS Sustain. Chem. Eng..

[cit59] Stosevski I. , Bonakdarpour A., Voon S. and Wilkinson D., Electrochemical Activation of Mn3O4 (Hausmannite) for a Rechargeable Aqueous Zn/Mn-Oxide Battery for Energy Storage Applications, Proceedings of the 2019 4th International Conference on Smart and Sustainable Technologies (SpliTech), Split, Croatia, 2019, pp. 1–5

[cit60] Lv H., Song Y., Qin Z., Zhang M., Yang D., Pan Q., Wang Z., Mu X., Meng J., Sun X., Liu X.-X. (2022). Chem. Eng. J..

[cit61] Ma X., Chen H., Ceder G. (2011). J. Electrochem. Soc..

[cit62] Palaniyandy N., Lakshmi D., Thenmozhi G., Kheawhom S., Musyoka N. N. (2024). J. Mater. Sci..

[cit63] Gong L., Zhang Y., Li Z. (2022). J. Electrochem. Soc..

[cit64] Chen H., Zhou W., Zhu D., Liu Z., Feng Z., Li J., Chen Y. (2020). J. Alloys Compd..

[cit65] Huang Z., Duan Y., Jing Q., Sun M., Tang B., Shi S. (2021). J. Alloys Compd..

[cit66] Long J., Yang Z., Yang F., Cuan J., Wu J. (2020). Electrochim. Acta.

[cit67] Zhou Z., Liu S., Wang J., Wu Y., Yang Y., Li Y., Wang J., Wang C. (2023). Appl. Surf. Sci..

[cit68] Sun M., Li D.-S., Wang Y.-F., Liu W.-L., Ren M.-M., Kong F.-G., Wang S.-J., Guo Y.-Z., Liu Y.-M. (2019). ChemElectroChem.

[cit69] Deng S., Xu B., Liu X., Yang Y., Xiao Y., Wang S., Zhao J., Chen T. (2025). Adv. Funct. Mater..

[cit70] Zhang Y., Liang J., Wu M., Tang Q., Xie Y., Li Z., Lu S., Qin L., Fan X. (2024). Mater. Today Commun..

[cit71] Wu Q., Fu Q., Mu C., Ao X. (2025). J. Alloys Compd..

[cit72] Syed W. A., Kakarla A. K., Yu J. S. (2026). J. Power Sources.

[cit73] Cao L., Chen M., Zhang Y., Hu J., Wu Y., Chen Y., Wang R., Yuan H., Wei F., Sui Y., Meng Q., Cheng L., Wang S. (2024). J. Colloid Interface Sci..

[cit74] Xu Y., Pan Z.-T., Wang Z.-C., Lu H.-P., Kong L.-B. (2025). ACS Appl. Energy Mater..

[cit75] Li Y., Yang Q., Wang J., Gao J., Wang X., Yang X., Hou H. (2026). J. Electroceram..

[cit76] Zhu S. Y., Yuan Y. F., Du P. F., Mo C. L., Cai G. C., Wang B. X., Guo S. Y. (2022). Electrochim. Acta.

[cit77] Stoševski I., Bonakdarpour A., Fang B., Voon S. T., Wilkinson D. P. (2021). Int. J. Energy Res..

